# Multidisciplinary approach to manage antenatally suspected placenta percreta: updated algorithm and patient outcomes

**DOI:** 10.1186/s40661-017-0049-6

**Published:** 2017-08-22

**Authors:** Paula S. Lee, Samantha Kempner, Michael Miller, Jennifer Dominguez, Chad Grotegut, Jessie Ehrisman, Rebecca Previs, Laura J. Havrilesky, Gloria Broadwater, Sarah C. Ellestad, Angeles Alvarez Secord

**Affiliations:** 1 Department of Obstetrics and Gynecology, Duke University Hospital, Durham, North Carolina 27710 USA; 2 Division of Gynecologic Oncology, Department of Obstetrics and Gynecology, Duke University Hospital, Durham, USA; 3 Division of Maternal Fetal Medicine, Department of Obstetrics and Gynecology, Duke University Hospital, Durham, USA; 4 Department of Interventional Radiology, Duke University Hospital, Durham, USA; 5 Cancer Statistical Center, Duke Cancer Institute, Durham, USA; 6 Duke Cancer Institute, Durham, USA; 7 Department of Anesthesiology, Duke University Hospital, Durham, USA

**Keywords:** Conservative management of placenta percreta, Delayed hysterectomy, Postpartum hemorrhage, Uterine artery embolization

## Abstract

**Background:**

Due to the significant morbidity and mortality associated with placenta percreta, alternative management options are needed. Beginning in 2005, our institution implemented a multidisciplinary strategy to patients with suspected placenta percreta. The purpose of this study is to present our current strategy, maternal morbidity and outcomes of patients treated by our approach.

**Methods:**

From 2005 to 2014, a retrospective cohort study of patients with suspected placenta percreta at an academic tertiary care institution was performed. Treatment modalities included immediate hysterectomy at the time of cesarean section (CHYS), planned delayed hysterectomy (interval hysterectomy 6 weeks after delivery) (DH), and fertility sparing (uterine conservation) (FS). Prognostic factors of maternal morbidity were identified from medical records. Complications directly related to interventional procedures and DH was recorded. Descriptive statistics were utilized.

**Results:**

Of the 21 patients with suspected placenta percreta, 7 underwent CHYS, 13 underwent DH, and 1 had FS with uterine preservation. Of the 20 cases that underwent hysterectomy, final pathology showed 11 increta, 7 percreta, and 2 inconclusive. 19/20 cases underwent interventional radiology (IR) procedures. Selective embolization was utilized in 14 cases (2/7 CHYS; 12/13 DH). The median time from cesarean section (CS) to DH was 41 [26–68] days. There were no cases of emergent hysterectomy, delayed hemorrhage, or sepsis in the DH group. Both estimated blood loss and number of packed red blood cell transfusions were significantly higher in the CHYS group. 3/21 cases required massive transfusion (2 CHYS, 1 FS) with median total blood product transfusion of 13 units [12–15]. The four IR-related complications occurred in the DH group. Incidence of postoperative complications was similar between both groups. Median hospital length of stay (LOS) after CHYS was 4 days [3–8] compared to DH cohort: 7 days [3–33] after CS and 4 days [1 –10] after DH. The DH cohort had a higher rate of hospital readmission of 54% (7/13) compared to 14% (1/7) CHYS, most commonly due to pain. There were no maternal deaths.

**Conclusion:**

This multidisciplinary strategy may appear feasible; however, further investigation is warranted to evaluate the effectiveness of alternative approaches to cesarean hysterectomy in cases of morbidly adherent placenta.

## Background

Placenta accreta includes various degrees of placental penetration into the myometrium that defines accreta, increta, and percreta. Placenta percreta is the most severe and least common form of placenta accreta (5–7% of cases) in which villi penetrate the entire myometrial thickness and reach or penetrate the serosa to involve adjacent organs. Although the specific incidence of placenta percreta is not known, the incidence of placenta accreta has risen over the past several decades likely due to the increasing cesarean delivery rate in this country, with a 10-fold increase in placenta accreta over the past 50 years [1]. By the year 2020, there are projected to be nearly nine-thousand cases of placenta accreta in the United States annually [2].

Abnormal placental attachment disorders are associated with a high rate of hemorrhage, coagulopathy, infection, urologic injury, and maternal death. The Society for Maternal-Fetal Medicine (SMFM) recognizes these risks and advocates that when clinical suspicion for placenta accreta exists, arrangements be made for delivery at an institution with appropriate expertise and facilities. Specifically, resources must be available for anticipated massive transfusion [3]. Despite advances in medical and surgical management of these patients, there continues to be an unacceptably high risk of maternal mortality, which is as high as 5.6% in women with placenta percreta [4].

There is no randomized study that evaluates cesarean hysterectomy compared to delayed hysterectomy (DH) in the setting of placenta accreta. Experience of delayed hysterectomy is obtained from case series of conservative approach to placenta accreta for women who desire fertility, whereby the placenta is left within the uterus, and *unplanned* hysterectomy is performed after cesarean delivery due to clinical deterioration [5, 6]. In the largest multicenter retrospective study of this conservative approach, 36/167 women required unplanned hysterectomy [7] 18/36 hysterectomies occurred within 24 h of delivery and all were the result of postpartum hemorrhage. The remaining 18 hysterectomies occurred after 24 h, with a median duration of 39 days (range 9–105 days). Reasons for delayed hysterectomy included hemorrhage, sepsis, uterine necrosis, vesicouterine fistula, arteriovenous malformation, and maternal request. Six percent (10/167 cases) had severe maternal morbidity with the most common causes due to sepsis and hemorrhage. There was one maternal death. Of these 10 severe morbidity cases, 8/10 underwent delayed hysterectomy and interestingly, the majority did not have severe placentation abnormality (only 2 percreta, 8 accretas). Of the 18/167 cases of percreta, severe maternal morbidity occurred in two. In these series, delayed hysterectomy was not planned since the primary intent was uterine conservation. Thus there is limited data on morbidity and outcomes related to *planned* delayed hysterectomy after delivery. Given the potential morbidity associated with delayed hysterectomy, only the morbidly adherent cases with potential extrauterine organ involvement should be considered as candidates.

Beginning in 2005, our institution has developed an algorithm for patients with suspected placenta percreta that involves integral communication and planning between multiple specialties. Our management algorithm has evolved since our initial reported cases [8] based on patient outcomes and ongoing experience. The objective of this study is to present our current strategy, maternal morbidity and outcomes of patients treated by our multidisciplinary approach, with careful attention to morbidity associated with planned delayed hysterectomy.

## Methods

After the Duke University Health System Institutional Review Board granted approval, retrospective identification for women with placenta percreta was conducted through SNOMED (Systematized Nomenclature of Medicine) diagnostic retrieval of the Duke pathology database. The search terms “Accreta,” “Increta,” and “Percreta” were utilized. The ICD-9 codes for these diagnoses were also used as search terms within the D.E.D.U.C.E. (Duke Enterprise Data Unified Content Explorer), an on-line research tool (http://deduce.duhs.duke.edu). All patients scheduled for elective DH from 2005 to 2011 were identified from the Gynecologic Oncology and Maternal-Fetal Medicine (MFM) operative scheduling logs. From August 2011 to 2014, patients with antenatally suspected placenta percreta were consented to participate in a prospective abnormal placentation database at Duke University Medical Center. Patients were identified during initial consultation with the gynecologic oncologist. For this study, only patients with a suspected diagnosis of placenta percreta since 2005 were included for analysis, given our contemporary approach began at this time.

Medical records were extracted for type of surgery performed, anesthetic technique, interventional radiology procedures utilized, suspected preoperative diagnosis by imaging, and final diagnosis based on pathology. Demographic data including age, race, gravidity, parity, body mass index, and number of prior cesarean deliveries were recorded. Prognostic factors of maternal morbidity such as duration of hospital stay, hospital readmission, administration of anti-fibrinolytic agents, use of cell salvage, blood transfusion, coagulopathy, activation of massive obstetric hemorrhage protocol, thrombosis, urologic injury, infection, unplanned intensive care unit (ICU) admission, sepsis, pulmonary edema, heart failure, bowel injury, and fistula formation were identified from medical records. We recorded complications related to specific treatment strategies including interventional procedures, methotrexate use, and DH. In addition, complications related to each treatment phase, including at time of cesarean section (CS), interval between CS and DH, and at time of DH, were recorded.

Descriptive statistics were used to analyze demographic and clinical characteristics. Statistical analysis was performed using SAS 9.3 (SAS Institute, Cary, NC). Wilcoxon rank sum tests were used to compare the number of blood transfusion units and estimated blood loss. Fisher’s Exact test (2-sided) was used to compare the total infection rates.

### Overview of multidisciplinary approach

**Fig. 1:**
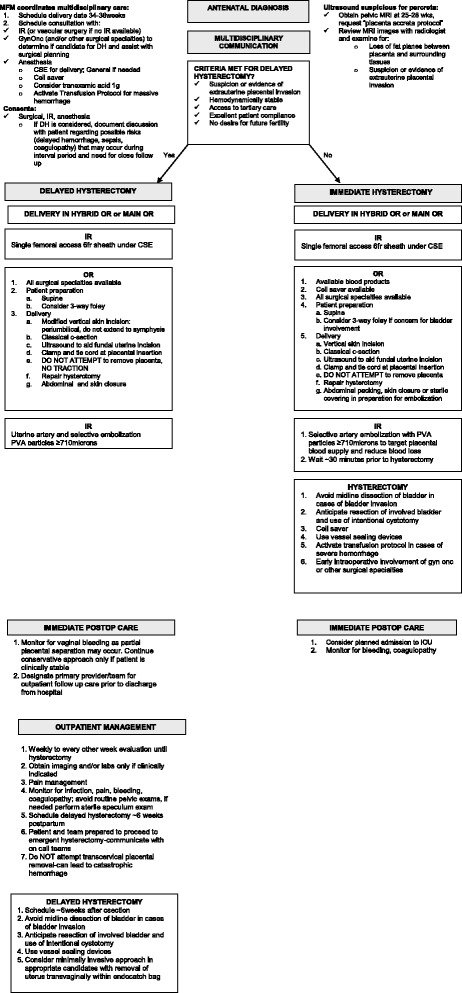
Algorithm for Placenta Percreta Management. Summary of considerations for each stage of treatment

Once antenatal diagnosis of placenta percreta is suspected on ultrasound, the MFM team coordinates communication and consultations with the Gynecologic Oncology, Interventional Radiology (IR), and Women’s Anesthesia divisions. Additional imaging with pelvic MRI is recommended to assist with surgical planning especially in cases with posterior or lateral placental involvement. The decision to proceed with immediate cesarean hysterectomy (CHYS) versus DH is an informed one between the patient and the multidisciplinary team. Criteria to offer the DH approach include: 1) prenatal imaging with ultrasound and/or MRI showing suspicion for placenta percreta with concern for extrauterine placental invasion or loss of planes between placenta and surrounding tissues; 2) no desire for future fertility; 3) clinical stability; 4) access to tertiary care center; and 5) patient willingness to comply with close follow up between delivery and interval hysterectomy. In the rare instance that patients desire future fertility, close expectant management occurs until placental resorption. Given the many steps and consulting services involved, a treatment algorithm has been developed at our institution (Fig. 1). This algorithm reflects our current practice at our institution and is updated from our initial approach in 2005.

### Delivery management

Delivery between 34 and 36 weeks gestation is performed in the hybrid operating room that has angiography capability. The recommendation for delivery during this gestational period is supported by a decision tree model that compared 11 strategies for optimal delivery timing in patients with vasa previa [9]. Our initial cases were performed in the main operating room (OR) and patients were transferred to and from the IR suite from the main OR for catheter placement and embolization after CS. Initially the patient came to the IR suite where bilateral femoral access was obtained and 7 French Flexor® Check-Flo® ANL2 sheaths (Cook® Medical, Bloomington In.) were placed and 8.5 mm occlusion balloons left in the internal iliac artery. Due to lack of use of the balloons and some CHYS not having embolization or balloon use, we converted to a single femoral artery 6 French vascular sheath to allow for selective embolization post-delivery by the IR team since 2011. Prophylactic balloon catheters are no longer utilized.

Neuraxial anesthesia remains the anesthetic of choice for cesarean delivery. Currently, neuraxial anesthesia is considered for delivery in both groups [10]. In these cases, it provides anesthesia for the placement of the femoral arterial sheath and the operative procedure, as well as post-operative analgesia. The interventional radiologist places the arterial sheath following placement of central venous access, arterial catheter and combined spinal epidural (CSE), if indicated. This order is necessary because the patient cannot be positioned for the neuraxial block placement after the sheath is in place. General anesthesia is performed in some cases based on patient preference and/or surgical indications, and should be considered for anticipated difficult airways.

The obstetrical team performs a modified midline periumbilical vertical incision that is *not* extended to the symphysis pubis and is made just large enough for delivery of the infant. For those cases in which the patient was deemed eligible for and desire DH, the final decision about whether to proceed with DH versus CHYS is made upon entry into the abdomen based on the intra-operative findings. A classical cesarean delivery is then performed in the awake parturient by a fundal incision under ultrasound guidance to avoid the placenta. The umbilical cord is ligated and transected at its placental insertion and the placenta is left undisturbed. Retraction on the cord is avoided. The uterine and abdominal incisions are closed in the usual fashion.

Intraoperative transfusion is at the discretion of the anesthesiologist, but we have had an institutional massive obstetric hemorrhage protocol in place since 2010 that can be activated to mobilize resources in these cases. We also have cell salvage available in the room for all cases. Recently, a tranexamic acid (TXA) 1000 mg slow intravenous bolus has been administered immediately after cord clamping.

Initially, bilateral uterine artery embolization was only performed for subjects who were having a DH. We now perform bilateral uterine artery embolization following delivery for both patients having an immediate CHYS and those having a DH. Bilateral uterine arteries and any branches recruited from other arterial distributions supplying the placental bed are embolized prior to removal of the groin catheter. Particle embolization ranging from 710 to 1000 μm up to 1000–1180 μm Contour™ polyvinyl alcohol (PVA) embolization particles (Boston Scientific Inc. Natick MA) are used until there is no visible supply to the placenta from the internal iliac artery. For subjects having an immediate hysterectomy, the embolization is performed following delivery while the abdomen is still open and just prior to hysterectomy. For women having a DH, the uterus and abdomen are first closed and then the patient undergoes embolization. The catheter is then removed from the femoral access site and the patient is transferred to recovery.

### Post-cesarean management

Prophylactic antibiotics for the retained placenta have not been administered routinely as our experience has not indicated benefit. Postpartum antibiotics are given when clinically indicated. Although used in our initial cases, methotrexate is no longer utilized. Patients are followed weekly to biweekly after cesarean delivery by the gynecologic oncologist until DH. Routine pelvic exams are avoided and are performed only when clinically indicated. For example, if patients present with increased vaginal bleeding and/or pain with the concern for partial expulsion of the placenta or infection. Transcervical removal of the placenta is avoided as this could lead to catastrophic hemorrhage. Pain management is emphasized and includes oral narcotics and anti-inflammatory medications until delayed hysterectomy. Laboratory and radiologic testing are performed only when clinically indicated. Routine imaging with either pelvic ultrasound or MRI is not performed prior to DH. Women desiring to breast feed are allowed and encouraged to do so during the period between delivery and DH.

### Delayed hysterectomy

The rationale for the six week interval between cesarean section and delayed hysterectomy is to allow time for return to normal maternal blood volume physiology, placental resorption, and uterine involution which would accommodate for transvaginal uterine removal for those cases having a laparoscopic approach to hysterectomy. The surgical technique to control the uterine arteries in the presence of an expanded lower uterine segment from the retained placenta has moved away from a modified radical hysterectomy approach with the use of vessel sealing instruments. However, caution should be used with these devices on thin walled placental vessels as shearing of the vessels can occur during attempted sealing or during release of the instrument. Laparoscopic approach has been performed in select cases with removal of the uterus transvaginally within a large endocatch bag.

## Results

Among 21 consecutive cases of antenatally suspected placenta percreta occurring between November 2005 and September 2014, seven underwent CHYS, 13 underwent DH, and one patient strongly desired future fertility and had successful uterine preservation. Given only the one case of uterine preservation, data is presented only for patients who underwent hysterectomy.

Nineteen of the 20 patients were diagnosed antenatally with imaging suspicious of placenta percreta and majority underwent both antenatal ultrasound and MRI (7/7 CHYS; 11/13 DH). There was one diagnosis of antenatal increta “cannot rule out percreta” on ultrasound and outside MRI did not confirm percreta. In this case, percreta was diagnosed at time of CHYS with visible placental involvement of the lower uterine segment. The median outpatient follow-up was 17 [0–108] days after CHYS and 55 [13–184] days after DH. Demographic and clinical characteristics are shown in Table 1. There were no differences between maternal age, gravidity, parity, gestational age, body mass index, or number of prior cesarean deliveries. In both groups, most had ≥2 prior cesarean deliveries (3/7 CHYS; 7/13 DH). The majority of patients had an additional prenatal placental diagnosis of placenta previa and/or vasa previa (6/7 CHYS, 13/13 DH). Forty-five percent (9/20) of cases were delivered on their scheduled date and the median gestational age at delivery was 35 weeks [18–37]. Reasons to deliver earlier included contractions, bleeding, fetal growth restriction, and premature rupture of membranes. There were no emergent cesarean deliveries performed. The final pathology revealed 11/20 increta and 7/20 percreta cases, while in 2 cases the diagnosis was indeterminate.

**Table 1:** Demographic and Clinical Characteristics

	Cesarean Hysterectomy *N* = 7 n (%)	Delayed Hysterectomy *N* = 13 n (%)
Maternal Age at Delivery, years	37 ± 3.7	31 ± 1.3
Maternal Race		
African-American	2 (29)	2 (15)
Hispanic	0	3 (23)
White	5 (71)	6 (46)
Other/Not Specified	0	2 (15)
Gravidity	4 [2–6]	3[2–8]
Parity	2 [1–3]	2 [1–4]
Gestational Age, weeks	35 [18–37]	34 [28–37]
BMI	28 [20–54]	30 [22–53]
Prior Cesarean Delivery		
1	4 (57)	6 (46)
2	2 (29)	6 (46)
3	1 (14)	1 (8)
Other Prior Uterine Surgery		
Uterine curettage	1 (14)	3 (23)
Hysteroscopic myomectomy	0	1 (8)
Concurrent Placental Diagnosis		
Partial previa	2 (29)	3 (23)
Complete previa	3 (43)	9 (69)
Vasa previa	1 (14)	1 (18)
Indication for Delivery		
Scheduled	3 (43)	6 (46)
Contractions	1 (14)	3 (23)
Vaginal bleeding	1(14)	1 (8)
Hematuria	1 (14)	1 (8)
Fetal growth restriction	1 (14)	1 (8)
Premature rupture of membranes	0	1 (8)
Final Pathology		
Accreta	0	0
Increta	4 (57)	7 (54)
Percreta	3 (43)	4 (31)
Inconclusive^a^	0	2 (15)

Data are n (%), mean ± mean standard error, or median with [range]

^a^Inconclusive pathology in two cases: 1) extensive degree of post-embolization myometrial infarction significantly hampered ability to recognize infiltrating chorionic villi and 2) changes in anterior myometrium may be consistent with prior placental involvement; however no retained placental identified

Table 2 summarizes surgical approaches, anesthetic, interventional radiologic and postoperative management. The decision to proceed to CHYS included no extrauterine involvement (3/7), patient preference (2/7), second trimester (1/7), and placental separation at time of CS (1/7). Initially general anesthesia was used for cesarean delivery in both groups. More recently, the majority of CS are now performed under neuraxial blockade with or without conversion to general anesthesia when indicated after delivery of the fetus (4/7 CHYS; 8/13 DH). Three patients received intraoperative TXA (2 CHYS, 1 DH). Intentional cystotomy to assess the necessary degree of bladder resection was performed in 3/20 cases (1/7 CHYS; 2/7 DH). All but one patient underwent an IR procedure. In both groups where prophylactic occlusion balloons were placed, none required inflation of the balloons to control hemorrhage. Prophylactic embolization occurred in 14 cases (2/7 CHYS and 12/13 DH). Only 2/13 DH cases received prophylactic IV antibiotics of 1–2 doses immediately after CS. Two patients were treated with extended antibiotics for endometritis and pyelonephritis, respectively. Three patients received weekly methotrexate with a median of 4 cycles given. There were no grade 3 or 4 toxicities related to methotrexate therapy. Methotrexate was last utilized in 2010. In the delayed cohort, the median time from cesarean delivery to delayed hysterectomy was 41 days [26–68]. Ten hysterectomies were performed via an open approach and three were performed laparoscopically, with two using the robotic platform.

**Table 2:** Management Strategies for Patients with Placenta Percreta

	Cesarean Hysterectomy *N* = 7 n (%)	Delayed Hysterectomy *N* = 13 n (%)
Reason to proceed to Cesarean Hysterectomy		
Placental separation occurred at time of delivery	1 (14)	N/A
No extra-uterine involvement	3 (43)	N/A
Patient preference	2 (29)	N/A
Second trimester	1 (14)	N/A
Anesthetic Technique at time of delivery		
General	3 (43)	5 (38)
Neuraxial	1 (14)	7 (54)
Neuraxial followed by general	3 (43)	1 (8)
Non-operative blood loss strategies		
Tranexamic acid (1 g)	2 (29)	1 (8)
Cell saver	2 (29)	0
Urologic Procedures		
Cystoscopy only	0	1 (8)
Ureteral stents	1 (14)	5 (38)
Intentional cystotomy	1 (14)	2 (15)
Interventional radiology procedures		
Prophylactic occlusion balloons only	3 (43)	0
Prophylactic occlusion balloon +embolization	1 (14)	7 (54)
Prophylactic embolization	2 (29)	5 (38)
Femoral access only	1 (14)	0
Prophylactic antibiotic after delivery for placenta left in situ	N/A	2 (15)
Methotrexate administration	N/A	3 (23)
Median number of cycles [range]		4 [4–5]
Interval of time (days) between delivery to hysterectomy	N/A	41 [26–68]
Surgical approach	N/A	
Modified radical		6 (46)
Total abdominal		4 (31)
Laparoscopic		3 (23)

Table 3 summarizes maternal morbidity at time of CHYS and for the DH cohort, at time of CS and at DH. 71% (5/7) of patients in the CHYS group required blood transfusion compared to 46% (6/13) in the total DH cohort. The median estimated blood loss was significantly higher in the CHYS cohort compared to both treatment phases of DH group, including at time of CS and at time of DH (2800 ml [400–4500] CHYS vs. 900 ml [400–1500] CS vs. 750 ml [50–2000] DH, *p* = 0.01). The median packed red blood cells (PRBC) transfusion was higher in the CHYS group compared to both phases in the DH group (2 [0–10] CHYS vs. 0 [0–3] CS vs. 0 [0–4] DH, *p* = 0.006). Massive transfusion (defined as greater than 4 units PRBCs) occurred in 2 cases (2 CHYS cases required 7 and 10 units, respectively) with a median total blood transfusion (including fresh frozen plasma, platelets, and cryoprecipitate) of 13 units [11–14]. Massive transfusion was not required in any of the DH cases. Although the infection rate was higher in the DH cohort (69% DH vs. 43% CHYS, *p* = 0.4), this was not significant and majority was due to urinary tract infections. The rate of coagulopathy, venous thromboembolism, and urologic injury were similar between all groups. Urologic injury included four cases of unintentional cystotomy (2 CHYS, 2 DH) and one ureteral injury in the DH group. There were no cases of unplanned ICU admission, fistula, bowel injury, pulmonary edema, or heart failure. In the DH cohort, there were no cases of delayed hemorrhage, sepsis, or emergent hysterectomy. There were no maternal deaths.

**Table 3:** Maternal Morbidity

	Cesarean Hysterectomy *N* = 7 n (%)	Cesarean section prior to Delayed Hysterectomy *N* = 13 n (%)	Delayed Hysterectomy *N* = 13 n(%)
Estimated blood loss	2800 [400–4500]	900 [400–1500]	750 [50–2000]
Total number of PRBC units transfused	2[0–10]	0[0–3]	0[0–4]
Total number of *patients* requiring PRBC	5 (71)	1 (8)	5 (38)
≤ 4 units	3 (43)	1 (8)	5 (38)
4 units	2 (29)	0	0
Infection			
Total	3 (43)	4 (31)	5 (38)
Endometritis	N/A	2 (15)	N/A
Wound Infection	3 (43)	0	2 (15)
Urinary Tract	0	2 (15)	2 (15)
Vaginal cuff Cellulitis	0	N/A	1 (8)
Coagulopathy^a^	2 (29)	0	2 (15)
Unplanned admission to the ICU	0	0	0
Venous thromboembolism	1 (14)	1 (8)	0
Urologic injury			
Total	2 (29)	0	3 (23)
Ureteral	0	0	1 (8)
Unintentional cystotomy	2 (29)	0	2 (15)
Interventional radiology complications	0	4 (31)	N/A
Paresthesia	0	1 (8)	
Transient hypoxia	0	1 (8)	
Ischemia (uterus)	0	1 (8)	
Gluteal ulcer	0	1 (8)	
Methotrexate toxicity	N/A	0	N/A
Spontaneous delivery of placenta prior to DH	N/A	1 (8)	N/A
Length of hospital stay (days)	3 [3–4]	4 [3–30]	4[1–10]
Hospital readmission	1 (14)	5 (38)	2 (15)
Wound infection	1 (14)	0	0
Pain	0	4 (31)	0
Bleeding	0	1 (8)	0
Ileus	0	0	1 (8)
Drainage of Uroma	0	0	1 (8)
Emergency department visit	1 (14)	2 (15)	0

Data are n (%) or median [range]

^a^Coagulopathy that required correction

Of the 19/20 hysterectomy patients who underwent interventional radiology procedures, there were no catheter-related injuries to the common femoral artery accessed for the procedure. Of the 14 total embolization procedures performed, none required re-embolization. The four possible IR-related complications occurred in the DH cohort where embolization was performed. One patient had transient paresthesia of the thigh, which may have been related to her embolization procedure or local anesthesia of the femoral nerve during access into the femoral artery. A second patient was found to have transient hypoxia during embolization. During the procedure, embolization with 700 to 900 μm Embospheres® (Merit Medical™, South Jordan, UT) was initially used. Following development of hypoxia, the decision was made to change to 710–1000 μm Contour™ PVA particles (Boston Scientific, Natick, MA) to reduce the potential for presumed shunting. It was difficult to determine if this was due to shunting or a pulmonary embolus. The patient subsequently was diagnosed with a pulmonary embolism. It is not clear if uterine artery embolization caused the pulmonary embolism. Following this case no additional hypoxic events occurred during embolization. This may have been due to converting to the use of 710–1000 μm Contour™ PVA particles (Boston Scientific, Natick, MA) or larger with the patients that followed this event. The last two possible IR related injuries include: 1) anterior uterine wall necrosis found on subsequent final pathology from hysterectomy and 2) an ulcer at the superior gluteal fold found approximately one month after delivery which may have been related to a radiation burn from fluoroscopy at time of procedure and/or nondirected embolization to this area.

The length of hospital stay (LOS) was similar between groups. However, there was one case in the DH cohort with an extended LOS of 33 days. This patient was not discharged after CS due to significant anemia and blood refusal status, as well as her long-distance residence. She remained inpatient receiving erythropoietin and IV iron per recommendations from our hospital’s Center for Blood Conservation until her hematocrit increased safely to proceed with delayed hysterectomy. The DH cohort had a higher rate of hospital readmission (including after CS and DH) as compared to the CHYS cohort (54% (7/13) vs. 14% (1/7)). In the DH cohort, pain was the most common reason for hospital readmission between delivery and delayed hysterectomy. One patient was readmitted during this interval period for observation secondary to bleeding but did not require transfusion.

## Discussion

With the rising incidence of abnormal placentation, it is important to define management strategies with a focus on reducing maternal morbidity and mortality. In the current study, we have shown that delayed hysterectomy may provide a feasible alternative to cesarean hysterectomy in patients with placenta percreta. Women with placenta percreta are known to have a high rate of hemorrhage, infection, urologic injury, and ICU admission despite treatment at a tertiary center [4]. Significant maternal bleeding is the most common complication, with an average reported estimated blood loss ranging between 3000 ml and 5000 ml [15]. Blood transfusion is required in 80–90% of cases, with large volume transfusion in 42% of cases [11–13]. Our delayed hysterectomy approach was associated with a significantly lower blood loss and transfusion rates. Furthermore, none of these patients required a massive transfusion. In our series, we demonstrate that planned DH occurring between four and six weeks following delivery is feasible. This duration of time allows for normalization of the hypervascular changes of the puerperal pelvis and therefore decrease the potential risk of massive hemorrhage and coagulopathy associated with cesarean hysterectomy [14, 16].

Methotrexate chemotherapy may increase the placental resorption rate, though not all investigators advocate for its use given that trophoblasts are not dividing in this setting [17–19]. Though rare, there can be severe adverse events such as immunosuppression and hepatotoxicity in patients who receive this medication. In our limited experience, we did not appreciate a significant clinical impact of methotrexate on the resorption of the placenta and therefore discontinued using this agent in 2010. Given the lack of data supporting the use of methotrexate we feel that further study is warranted prior to routine use, especially since it is contraindicated with breast feeding.

Our approach utilizes prophylactic uterine artery embolization (UAE) to reduce the risk of post-delivery hemorrhage while the placenta remains in situ. Initially, the rationale for *prophylactic* UAE was supported by higher success rates of UAE when used in a non-emergent setting [20]. This has been again demonstrated in a recent series that showed embolization failure was associated with disseminated intravascular coagulation and transfusions of more than ten units [21]. Furthermore, Angstmann et al. demonstrated a statistically significant reduction in blood loss in patients who underwent embolization prior to hysterectomy when compared to those who underwent immediate cesarean hysterectomy without embolization [11]. Thus, we have recently incorporated this into our algorithm when CHYS is planned for placenta percreta cases. The decreased transfusion requirements in our DH cohort may be related to prophylactic UAE, which was performed in 12/13 DH cases compared to only 2/7 CHYS cases. Prophylactic UAE may be helpful in decreasing potential delayed hemorrhage and may contribute to placental resorption in the period between delivery and delayed hysterectomy. However, there is no evidence to support or refute the use of prophylactic UAE when the placenta is left in situ and further investigation is required.

Prophylactic placement of arterial balloon-occlusion catheters in the anterior divisions of the internal iliac arteries can provide temporary hemostasis if severe hemorrhage is encountered during cesarean hysterectomy [22, 23]. However, previous studies have shown no impact of balloons on risk of transfusion [24]. Other investigators have found an unacceptably high rate of complications related to catheter placement (including artery thrombosis and dissection) and recommended against the use of prophylactic intravascular balloon catheters [25, 26]. In our series, occlusion-catheters that were placed in 12 women prior to delivery were only inflated in the fertility-sparing case. Thus, our current algorithm has moved away from routine placement of balloon occlusion catheters.

Patients have a consultation with an obstetric anesthesiologist prior to surgery to discuss anesthetic options, and are routinely offered neuraxial anesthesia for the cesarean delivery in both CHYS and DH approaches. Neuraxial anesthesia is the preferred mode of anesthesia for obstetric patients, as it is associated with fewer airway complications and less bleeding than general anesthesia [27, 28]. Many patients wish to remain awake to experience the birth of their child, and we do allow a support person to remain in the room with the patient until the birth of the baby. Light sedation is often employed for placement of lines and interventional radiology groin access prior to delivery. In some cases that required massive transfusion, general anesthesia was induced after the birth of the infant.

Non-operative blood conserving strategies such as pre-operative treatment of anemia and the use of cell saver, should be considered at time of delivery and hysterectomy [29, 30]. Acute normovolemic hemodilution was utilized for both delivery and hysterectomy in our one case of blood refusal and is described elsewhere in detail [31].

The antifibrinolytic agent, tranexamic acid (TXA), has been shown to decrease the need for blood transfusion in a wide range of planned surgeries, without an increased risk of thrombotic events, [32] and to decrease mortality in bleeding trauma patients [33]. More recently it has been described for the management of obstetric hemorrhage [34] and is currently under investigation in a randomized clinical trial [33]. Although further study is needed to confirm the use and safety of TXA in this population, we have incorporated low-dose TXA (1 g) as a standard part of the protocol for these cases. This dose has been used in a number of trials for the prevention of postpartum hemorrhage, and few adverse events have been reported [32]. However, none of the randomized-controlled trials that have been conducted to date have reported neonatal outcomes, and TXA does cross the placenta. Therefore, until more robust, larger trials confirm neonatal safety, we have chosen to administer TXA after cord clamping.

Our study is limited by its retrospective nature, duration of the study, and its small sample size. Although the numbers of placenta percreta cases are likely increasing, the low incidence is a barrier to conduct prospective randomized controlled studies. An important limitation is our inability to assess the relative value of each component of our algorithm for women with placenta percreta. For example, “What is the benefit of prophylactic UAE relative to awaiting placental resorption and delaying hysterectomy?” However, our algorithm is undergoing real-time assessment and continues to mature based on our clinical experience and hopefully will help us address these important questions. Other considerations that were not measured in this study are the potential psychosocial impact of undergoing a second surgery in the postpartum period and cost effectiveness of treatment. Discussion of accurate antenatal diagnosis of placenta percreta is beyond the scope of this study; however, given our experience we have recommended pelvic MRI to be performed at our institution and reviewed by our interventional radiology team. Finally, all of our patients were treated at the same tertiary care center with strict criteria to offer the delayed hysterectomy approach and thus our results may have limited generalizability.

It is important to emphasize that multidisciplinary care for a rare condition requires appropriate coordination. Identifying an interested faculty attending, rather than a trainee, from each specialty to be the point person to contact for each case is imperative. As these cases will likely be treated in larger academic centers where trainees are involved, we have learned that because of trainee turnover and infrequency of cases, primary coordination by a designated MFM attending, involvement of consistent faculty from each specialty, and having a written protocol are critical to avoid inconsistencies and maximize patient outcomes.

## Conclusion

We present our current multidisciplinary management strategy for those select patients with the most severe forms of abnormal placentation. The maternal morbidity and outcomes of these patients reflect that uterine artery embolization with delayed hysterectomy appears to be feasible. Given the significant morbidity and mortality of placenta percreta cases, further study is warranted to investigate potential alternatives to cesarean hysterectomy for those women who are at highest risk of maternal morbidity.

## Abbreviations

CHYS: Immediate hysterectomy after cesarean section
CS: Cesarean section
CSE: Combined spinal epidural
DH: Delayed hysterectomy
FS: Fertility sparing
IR: Interventional radiology
LOS: Length of stay
MFM: Maternal fetal medicine
OR: Operating room
PRBC: Packed red blood cells
PVA: Polyvinyl alcohol
TXA: Tranexamic acid
UAE: Uterine artery embolization
